# Optimization and Parallelization of Sorting by Interfacial Tension (SIFT) for High-Throughput Metabolic Cell Sorting

**DOI:** 10.3390/mi17060691

**Published:** 2026-06-03

**Authors:** Aria Trivedi, Thomas Mathew, Matthew Shulman, Lakshmi Thangam, Pooja Dubey, Charlotte V. Cohen, Kelsey Voss, Paul Abbyad

**Affiliations:** 1 Department of Chemistry and Biochemistry, Santa Clara University, Santa Clara, CA 95053, USA; ariatrivedi@gmail.com (A.T.);; 2 Department of Pharmacology, University of Virginia, Charlottesville, VA 22903, USA

**Keywords:** microfluidics, droplet microfluidics, cytometry, sorting, passive sorting, metabolism, glycolysis, cells, T-cells

## Abstract

A systematic optimization of throughput and operational run time in Sorting by Interfacial Tension (SIFT) is presented. Reducing droplet size and enabling a broader distribution of droplet trajectories increased the number of droplets processed per sorting element, resulting in about a four-fold improvement in throughput from 30 to 125 droplets per second. Throughput was further enhanced through device parallelization, as demonstrated by devices incorporating two and four independent sorting regions. These configurations distribute droplets evenly across sorting elements. The elements exhibited comparable pH sorting thresholds, indicating similar flow conditions and drag forces within each region. Among the designs evaluated, the two-element configuration provided the optimal balance of throughput, specificity, robustness, and simplicity. It achieved maximum throughputs of about 250 droplets per second. In many biological applications, only 1 in 20–30 droplets are occupied to minimize multiple-cell occupancy, resulting in an effective sorting rate of approximately 8 cells per second. Throughput and pH sorting thresholds were preserved for two hours of continuous cell sorting. The improved platform was applied to examine the relationship between cellular glycolysis and iron homeostasis at the single-cell level in activated Jurkat cells. It revealed a subpopulation of highly glycolytic cells with significantly elevated iron uptake, consistent with prior reports linking iron regulation and T cell metabolism. Collectively, these advances expand the scale, stability, and biological applicability of SIFT. These advances facilitate large-scale functional studies and the capture of rare, metabolically distinct, cell populations.

## 1. Introduction

The separation of cell populations is essential in modern cell biology and medicine. The primary workhorse for this task is Fluorescence-Activated Cell Sorting (FACS), which typically sorts cells via a fluorescence probe. Although expensive, it offers high throughput and unmatched versatility. However, FACS ultimately depends on the availability and specificity of suitable probe molecules to identify target populations, which limits its applicability for some separations. Furthermore, it is not readily amenable to sorting live cells directly based on their secretions or metabolic activity. Label-free microfluidic approaches, including acoustophoresis [1], dielectrophoresis [2], inertial microfluidics [3,4], and deterministic lateral displacement [5,6], enable inexpensive, high-throughput cell sorting. These techniques separate cells by their size, density, deformability and electrical properties. However, many applications require the separation of cells based on cellular activity rather than physical characteristics. This is especially important for sorting cells that share similar physical characteristics, such as isolating a subpopulation of cells with distinct metabolisms from within a group of cells of the same type.

Droplet microfluidics has become a powerful and versatile tool for the biological analysis of individual cells. This is largely because cellular secretions remain confined within the droplet and therefore remain associated with the originating cell. This enables the integration of high-throughput droplet sorting of target cells with cell recovery and downstream analysis, including transcriptomic, proteomic, and metabolomic profiling [7,8,9].

Our lab has developed a droplet microfluidic technique, Sorting by Interfacial Tension (SIFT), that allows for the passive and label-free sorting of droplets by pH. The technique differentiates itself by its breadth of applications as it can sort enzymes [10], cells [11,12,13] and amplified DNA [14]. As acidification of the extracellular space is linked to a cell’s glycolysis via lactate and proton secretion, it is especially versatile for the sorting of cells based on metabolic activity. It has been used to separate empty vs. cell occupied droplets, live vs. dead cells, cancer vs. non-cancer cells, naive vs. activated T cells, and cells treated with a pharmaceutical vs. non-treated cells [11,12,13]. The technique is particularly well suited for separating cells of the same type. For example, it can isolate cancer cells with high glycolytic activity [12], a cell characteristic associated with malignancy [15,16], thereby isolating an important subpopulation of cells that could be targeted therapeutically.

Despite its versatility and utility, the limited throughput of SIFT has constrained its use in certain applications. Many cellular assays are either facilitated by or require large numbers of cells. For example, downstream analyses, such as bulk RNA sequencing and mass-spectrometry-based metabolomics, typically require pooling large numbers of cells [17,18]. Moreover, the ability to sort large numbers of cells is essential for isolating rare populations such as antigen-specific activated effector T cells or circulating tumor cells. These cells occur at very low abundance yet often exhibit distinctive glycolytic metabolic states [4,19].

The throughput of our first iterations of SIFT devices was approximately 30 droplets per second [11,12]. To minimize multiple-cell occupancy, cell density is often adjusted so that only one in every 20–30 droplets contain a cell, leading to an effective sorting rate of approximately one cell per second. A similar device used to sort amplified DNA droplets also achieved throughputs of tens of droplets per second [14]. A higher throughput (~70 droplets per second) was reported in our most recent paper [20]. This paper incorporated one of the changes described here, specifically the use of smaller droplets. Although other interfacial-tension-based sorting devices have demonstrated higher sorting rates (up to 250 Hz) [21], direct comparison is difficult because those systems did not sort based on pH or cell glycolysis. The higher throughputs reported in those studies may therefore not reflect differences in the device. Instead, they may arise from the larger interfacial tension between the specific aqueous samples and the continuous oil phase used, potentially enabling higher flow rates without compromising sorting accuracy [21].

Here, we describe a systematic increase in the total number of cells sorted by (i) optimizing the droplet size and path, (ii) parallelizing the sorting region, and (iii) increasing the device run time. Taken together, these modifications increase the sorting throughput by several-fold, reaching ~250 droplets per second or about 8 cells per second. These improvements extend the operational run time by a factor of six.

As a biological demonstration of the improved device and its integration into a broader biological workflow, single-cell intracellular iron levels were measured in activated T cells separated according to high and low glycolytic activity. T cells play a central role in the adaptive immune response. Iron has been shown to enhance glucose metabolism and glycolysis in immune cells, including T helper cells, through activation of metabolic reprogramming pathways such as AKT–mTOR and upregulation of the glycolytic regulator PFKFB4 [22,23]. However, the interplay between glycolysis and intracellular iron during early T cell activation remains poorly understood. We demonstrate here that some highly glycolytic T cells isolated by our device exhibited elevated intracellular iron levels. Improvements in device throughput and run time significantly broaden the scope of investigations that can be addressed using SIFT.

## 2. Materials and Methods

Cells: K562 human chronic myelogenous leukemia cells were grown in ATCC-formulated Iscove’s Modified Dulbecco’s Medium (IMDM) and Jurkat Clone E6-1 TIB-152™ Human Acute T cell leukemia cells were grown in ATCC-formulated RPMI-1640 Medium. Both cell lines were purchased from ATCC (Manassas, VA, USA) Cells were grown at 37 °C in a 5% CO_2_ atmosphere supplemented with 10% fetal bovine serum (HyClone, GE Healthcare Life Sciences, Logan, UT, USA) and 2% *v*/*v* penicillin–streptomycin (10,000 units/mL–10,000 μg/mL) solution (Gibco, Life Technologies Corporation, Grand Island, NY, USA).

Cell preparation for on-chip experiments: Jurkat cells were activated with soluble activation complexes (ImmunoCult, StemCell Technologies, Vancouver, BC, Canada) following the manufacturer’s protocol and were incubated for 24 h. This initial activation step was not performed for K562 cells. On the day of the experiment, cells were centrifuged, washed, and resuspended in buffer (HBBS for Jurkat cells and PBS for K562 cells). To better identify droplet occupancy, K562 cells were labeled by incubating them for 30 min at 37 °C and 4% CO_2_ atmosphere with Calcein AM (Thermo Fischer, Waltham, MA, USA), a viability fluorescent dye. Alternatively, for the iron assay on Jurkat cells, the cells were labeled with BioTracker Far-red Labile Fe^2+^ Live Cell Dye (MilliporeSigma, Burlington, MA, USA) at 5 µM solution in HBSS for 30 min at 37°C at 5% CO_2_. Calcein AM was not used when staining with BioTracker to avoid spectral overlap in the fluorescence signals. Subsequently, the cells were washed again and resuspended in on-chip solutions at a cell concentration of 1.0–1.5 × 10^6^ cells/mL, which was determined using a Cellometer Auto T4 Bright Field Cell Counter (Nexcelcom Bioscience LLC, Lawrence, MA, USA). The cell solution is kept dilute to avoid multiple cell occupancy in a droplet. The cell solution was then filtered through a Flowmi 40 µm cell strainer (MilliporeSigma, Burlington, MA, USA) to ensure debris was removed from the solution and to reduce cell clumps. On-chip solutions were a 1:1 mix of media and 1.5 mM PBS buffer. The media was prepared without fetal bovine serum (deproteinated media), both solutions were supplemented with 1% *w*/*w* Pluronic F-68 (Affymetrix Inc., Maumee, OH, USA), 15% *v*/*v* Optiprep solution (Fresenius Kabi Norge AS for Axis-Shield PoCAS, Oslo, Norway) and 0.1 mg/mL pyranine (AAT Bioquest Inc., Sunnyvale, CA, USA). The solution pH and osmolality (determined with Vapro Vapor Pressure Osmometer 5520, Wescor, ELITech Biomedical Systems, Logan, UT, USA) of on-chip solutions were adjusted to physiological values (pH 7.4–7.6; 280–320 mOsmol) prior to the experiment. Pluronic F-68 was used to promote droplet stability and cell viability, whereas Optiprep modulated solution density to limit cell sedimentation within the syringe, tubing and droplets.

Microfluidic device: Chips with channel depth modulations were fabricated from polydimethylsiloxane (PDMS), utilizing the dry-film photoresist soft lithography technique previously reported by Stephan et al. [24]. This technique facilitates easy prototyping with multilevel designs. The PDMS chip was irreversibly bonded to a glass slide via plasma treatment. To render the internal surfaces of the channel hydrophobic, the channels were treated with Novec 1720 electronic grade Coating (3M, Maplewood, MN, USA) for 30 min at 150 °C. Channel geometry and dimensions for the device with two sorting regions are shown in Figures S1 and S2, respectively, while those for the device with four sorting regions are provided in Figures S3 and S4.

Droplet sorting and measurements: The general use of the sorting device was similar to what has been described previously [11,13] and is described here for the device with two sorting regions. Briefly, the chip consists of a droplet generator where cells are encapsulated into droplets; an incubation channel enabling a change in droplet pH due to the cells’ metabolism; and a sorting region. Cellular solution was injected into the chip through an aqueous inlet. Via a flow focuser, droplets were generated in 0.1% *w*/*w* Picosurf-1 surfactant oil (Sphere Fluidics Limited, Cambridge, UK) in Novec 7500. An additional oil outlet after droplet generation was set to flow in the opposite direction of the main flow to reduce the amount of oil before the droplets entered the incubator region. This enabled tight packing of droplets within the incubator to ensure the same incubation times for all droplets [25]. The length of the incubation channel was 10 cm. Incubation time ranged from 3 to 6 min depending on the experiment before the channel narrowed and droplets entered the sorting region. At the end of the incubator, oil solution, QX100 droplet generation oil for probes (Biorad, Hercules, CA, USA), entered the chip through two inlets, the QX100 inlet and the Oil Entrainment Inlet (Figure S1). This oil/surfactant combination is called here QX100 for simplicity and consistency with prior publications. Droplets entered the two sorting regions, which both had the same dimensions and flow. The rails, oriented at 45 degrees to the flow direction, allowed droplets to be sorted by interfacial tension and hence pH. Selected and unselected populations from both of the sorting areas were combined before they entered the collection outlets.

Flows within the chip were controlled via a computer-controlled syringe pump system (Nemesys, Cetoni, Korbussen, Germany). A very small stir bar (2 × 2 mm) was placed inside the 0.5 mL cell solution syringe. A large external magnet was used to gently mix the solution every 10 min to prevent cell sedimentation. PEEK tubing (inner diameter: 180 µm) connected the cell solution syringe to the chip (VICI Precision Sampling, Baton Rouge, LA, USA). Typical flow conditions can be found in the Supplementary Materials, Table S1. The temperature of the chip during experiments was maintained at 37 °C using a heating stage with a control module and temperature feedback (CHS-1 heating plate, TC-324C temperature controller, Warner Instruments, Hamden, CT, USA).

On-chip images and videos were taken on an inverted fluorescence microscope (Olympus IX-51, Center Valley, PA, USA) equipped with a 4X objective, a shuttered LED fluorescence excitation source (Spectra-X light engine, Lumencor, Beaverton, OR, USA) and a high-speed camera (VEO-410, Vision Research, Wayne, NJ, USA). The microscope filter cube contained a dual-edge dichroic mirror (Di03-R488/561-t1-25 × 36, Semrock, IDEX Health & Science LLC, Rochester, NY, USA) and dual-band emission filter (FF01−523/610-25, Semrock) that enabled transmission of pyranine and Calcein AM fluorescence. The excitation source with individually addressable LEDs was coupled to an Arduino (Arduino LLC, Scarmagno, Italy) to enable rapid alternation between LED colors through simple TTL triggering for determining droplet pH values. Droplets were excited with alternating violet (395 nm BP 25 nm), blue (440 nm BP 20 nm) and green excitation (561 nm BP 14 nm) at a rate of 100 frames per second (33 fps for each color) for pyranine pH measurements. For long sorting experiments, 2 min videos were taken about every 10 min.

Cell collection: Cell collection was performed using a workflow first described in Shulman et al. [20] and summarized in Figure S5. Cells were first sorted as described above and collected into 1 mL pipette tips, inserted directly into the chip outlets. The pipette tips were prefilled with 300 µL of 0.1% *w*/*w* Picosurf-1 in Novec 7500 to dilute the surfactant found in QX100. Minimizing exposure to this acidic surfactant was found to improve cell viability. An aliquot of 300 µL of HBSS droplets (diameters of 50–200 μm) made in 0.1% *w*/*w* Picosurf-1 in Novec 7500 was also added to the pipette tips. The empty droplets improved cell recovery by ensuring that sorted cell droplets did not collect on the pipette walls and facilitated droplet coalescence. To avoid overflowing the pipette tip over the course of the experiment, oil was removed every 10 min from the pipette tip using a long blunt needle syringe. Care was taken during the removal of oil to avoid disturbing the chip or provoking the coalescence of the droplets layered above the oil. After each oil removal, 100 µL 0.1% *w*/*w* Picosurf-1 in Novec 7500 was added to the pipette tip to improve post-sorting cell viability. The pipette tips were capped between transfers to avoid possible contamination.

At the end of the sorting experiment, pipette tips were removed and the oil was drained from the bottom of the tip. Droplets were then collected into microcentrifuge tubes. Droplets were coalesced using a static gun (MILTY Pro Zerostat 3, Goldring, Bishop’s Stortford, UK) [26]. To assist in droplet coalescence, Novec 7500 was added to the microcentrifuge tubes during the coalescence process. Once the droplets were coalesced, 20 µL of each cell solution was transferred into individual wells of a 96 well fluorescence plate. To sediment the cells to the bottom of the plate for imaging, the plate was centrifuged at 1500 rpm (g-force 525) for 5 min using a TX-100 swinging bucket rotor at 20 °C in a Sorvall Legend XTR Centrifuge (Thermo Fisher Scientific, Waltham, MA, USA).

Data analysis: ImageJ software version 1.54p was used for image analysis [27]. pH values of individual droplets were determined at the end of the incubation channel before droplets entered the sorting region via the ratio of fluorescence intensity of pyranine from the background-subtracted ratio of two-color excitation. A calibration curve from fluorescence ratios of blue and violet excitation for droplets of known pH was used to determine pH, using a procedure described previously [12]. Green excitation was used to identify cells labeled with Calcein AM. In one experiment, the rail position within the bottom sorting regions led to some droplets missing the rail entirely. In this case, droplets that did not encounter the rail represented less than 5% of the total and were excluded from the analysis. Logistic regression was used to statistically estimate optimal pH thresholds to separate selected from non-selected cells. More specifically, the form, logit(P) = β_0_ + β_1_x where logit = ln((P)/(1 − P)) was used. β_0_ and β_1_ were optimized when performing the fit, representing the intercept and slope, respectively. The pH threshold was defined at a 50% predicted probability of selecting the cell. The standard error of the prediction was used to obtain a 95% confidence interval around that threshold.

Collected cells were imaged with an inverted fluorescence microscope (Olympus IX-50, Center Valley, PA, USA) equipped with a 20X objective, a shuttered LED fluorescence excitation source (Sola SE-II), Lumencor, Beaverton, OR, USA) and a CMOS camera (Orca Flash 2.8, Hamamatsu, Bridgewater, NJ, USA). Fluorescence images were obtained with long exposure (1.9 s) using a red fluorescence cube (Ex: 631 nm BP 28 nm, Dichroic mirror 652 nm, Em: 680 nm BP 42 nm). Debris and irregularly shaped cells were excluded from the analysis. Maximum cellular fluorescence was measured in ImageJ as the dye is expected to be localized in the endoplasmic reticulum based on the manufacturer’s documentation.

## 3. Results and Discussion

SIFT allows for the passive and label-free sorting of cells based on cell glycolysis. The technique leverages a dependence on the droplet interfacial tension with pH in the presence of a surfactant. Cell glycolysis is coupled with a secretion of protons, leading to a decrease in droplet pH. This enables the selection of droplets by interfacial tension and, hence, single-cell glycolysis.

**Figure 1:**
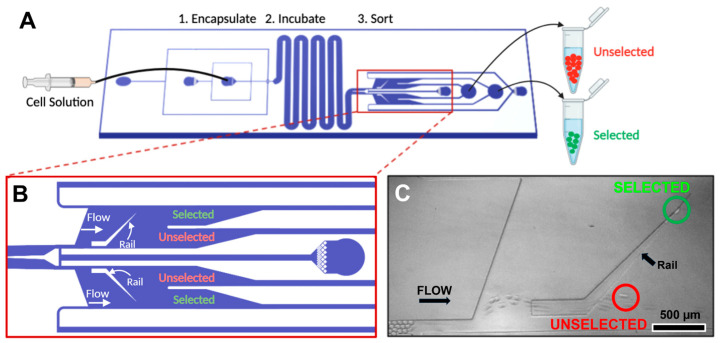
(**A**) Schematic of SIFT chip device for sorting a population of cells with low (red) and high (green) glycolysis. Created in BioRender.com. (**B**) Chip geometry of device with two sorting regions (**C**) Image of sorting showing a droplet containing a cell with high glycolysis ride the rail laterally up (circled in green). Empty droplets or those containing cells with low glycolysis (circled in red) are only slightly deflected by the rail.

Figure 1A shows the basic chip geometry of the current SIFT device, which integrates cell encapsulation, incubation, and sorting into a single continuous-flow inline platform. In many other droplet sorting devices [7,21,28], these steps are decoupled. Cells are encapsulated into droplets in one device, incubated off-chip and then droplets are injected into another device for sorting. Integrating these steps within a single device eliminates user intervention between stages and reduces the potential failure points associated with droplet collection and reinjection. It also simplifies setup, as only one chip needs to be prepared. Briefly, the device operates as follows. Cells are encapsulated into droplets, with the cell concentration kept low to avoid multiple cell occupancy in a droplet. Droplets then flow through a long serpentine incubation channel. During this time, cell glycolysis will lead to the acidification of the droplet. Lastly, droplets enter the cell-sorting step. Figure 1B shows a detailed view of the sorting geometry (channel geometry and dimensions of sorting region are provided in Figures S1 and S2, respectively). In this diagram, a channel geometry with two parallel sorting regions is presented.

As droplets enter the sorting step, a carrier oil solution (QX100) is introduced, which exhibits an inverse relationship between droplet pH and interfacial tension [11]. Droplets, flattened by the top and bottom of the channel, enter the rail region of increased channel height. The droplets are less confined in the rail and therefore adopt a more spherical shape to minimize their surface energy. Droplets of high pH, and hence low interfacial tension, are pushed off the rail by the oil entrainment flow and directed to an unselected chip outlet (Figure 1C). Droplets of low pH, and hence high interfacial tension, remain in the rail as the entrainment flow is insufficient to re-confine the droplets. The droplets follow the rail, oriented 45 degrees relative to the direction of flow, and exit the tapered rail near the top. These droplets are directed towards the selected chip outlet. In this way, cells with high or low levels of glycolysis are separated and directed towards different chip exits for recovery.

The flow conditions on the chip can be adjusted to control pH selection, as droplet selection depends on the hydrodynamic drag in the rail region [12]. The entrainment flow therefore provides a user-controlled parameter to tune droplet pH selection with higher flows decreasing the pH of selection. The pH of droplets after incubation depends on cell type and treatment. Accordingly, the entrainment flow is adjusted during the experiment to obtain the desired fraction of selected cells. In the experiments presented here, the flow was typically set to select approximately half of the cells.

Early iterations of the SIFT device [11,12] achieved sorting throughputs of approximately 10–30 droplets per second. The device could be run continuously for 10–20 min. As most droplets are empty (typically 1 in 20–30 droplets contain a cell), this effectively meant the sorting of several hundred cells. Here, we describe a series of optimizations, which are presented in separate sections covering droplet size and path, parallelization, and run time. These optimizations lead to typical throughputs of 200–250 droplets per second and a sorting run time of 2 h. Collectively, these improvements enable the sorting of several thousand cells. The device is also simpler, requiring five independent syringe pumps rather than the six used by previous devices [12,13].

### 3.1. Optimizing Droplet Size and Path

A direct way to increase droplet throughput is by increasing the number of droplets that are sorted by an individual sorting element. Several changes were made to increase the sorting throughput. The first was to decrease the droplet size from 70–90 µm to 40 µm. This was achieved by decreasing the nozzle width of the flow focuser from 50 µm to 30 µm.

Using smaller droplets has several implications for both the function and the sorting in the device. The smaller volume means that droplets change pH quicker due to cell glycolysis. Consequently, the incubation time could be short, at 3–6 min, while still maintaining a pH change suitable for sorting. This allows faster overall flow rates in the incubator. Moreover, due to the smaller size, the overall number of droplets in the incubator can be increased. This results in more droplets reaching the sorting step each second and ultimately sets an upper limit for the sorting rate.

**Figure 2:**
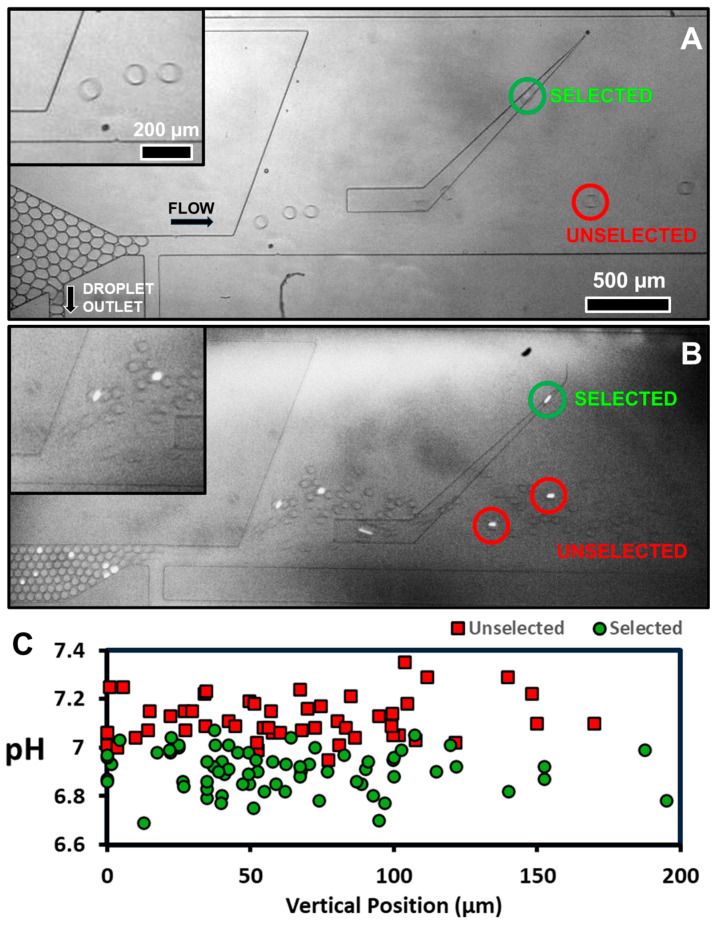
(**A**) Image of sorting region for previous SIFT sorting devices. An unselected droplet (circled in red) and selected droplet (circled in green) are highlighted in the image. Large droplets (70–90 µm diameter) follow a similar path in the sorting region. Cells appear as bright spots due to fluorescent labeling. Inset: (upper left) shows an enlarged view of the beginning of the sorting region. (**B**) Image of sorting region of current SIFT sorting device. Smaller droplets (40 µm diameter) follow different paths in the sorting region. (**C**) pH of unselected droplets (*N* = 53) (red squares) and selected droplets (*N* = 71) (green circles) plotted as a function of the maximum vertical position reached by the droplet in the sorting region.

In previous iterations of the device, an effort was made to ensure that droplets entered the sorting region one by one and followed a similar path (Figure 2A). A similar approach is seen in other papers [14]. This also avoided droplet collisions that may impact specificity. To ensure well-spaced droplets, an additional outlet (labeled droplet outlet in the bottom left of Figure 2A) with a negative flow removed droplets before they entered the sorting region [11,12]. In most cases, approximately half of the droplets were removed from the device prior to sorting. This throttled the number of droplets entering the sorting region, ensuring a consistent droplet path. Although this approach yielded strong pH specificity, it ultimately constrained the device’s throughput.

An alternative approach is to increase the number of droplets entering the sorting region at the end of the incubation stage. At higher droplet densities, inter-droplet interactions become significant, leading to droplet collisions. Droplets will take different paths due to collisions between droplets. In addition, droplet trajectories vary due to local changes in hydrodynamic resistance induced by neighboring droplets [29]. These interactions have been used to direct sequential droplets to alternating branches of narrow channels [30,31]. The dynamics of densely packed droplets entering a wider channel, as occurs when droplets enter the sorting region, have been less extensively studied in microfluidic systems. However, in this case, droplet interactions would lead to a spread of droplet trajectories as droplets compete for available flow paths in the expanded geometry [32]. Figure 2B shows the end of the incubator and sorting region for the current implementation of the device. Upon entering the sorting region, droplets scatter from the outlet and take different paths in the sorting region. Based on the droplet dimensions and available paths, roughly three droplets could be spaced to flow laterally in parallel. However, in practice, the droplets were typically staggered in the sorting region. In this new design, all droplets enter the sorting region and no droplets are discarded. This change was found to increase device stability while increasing throughput to 100–125 droplets per second. It also reduced the number of syringe pumps required to operate the SIFT device from six to five.

The pH selection can be studied as a function of the different paths. Figure 2C shows the selection of droplets based on the maximum lateral, or vertical, position on a droplet’s path. A pH threshold is defined as the average pH of droplet selection and is determined by a fit to a logistic regression. By comparing the boundary between selected and unselected droplets, it is observed that the threshold is largely maintained, regardless of the path. The threshold for all droplets in Figure 2C was determined to be 7.02 ± 0.01 (95% confidence interval) (Figure S6). When the threshold was instead evaluated at different vertical positions (0–50 μm, 50–100 μm, and >100 μm), the corresponding thresholds were 7.03 ± 0.01, 6.99 ± 0.02, and 7.03 ± 0.02, respectively (Figure S7). These similar thresholds indicate that droplet path and inter-droplet collisions do not appear to substantially impact pH selection.

Based on the results in Figure 2C, the percent sensitivity and specificity can also be evaluated to determine the accuracy of sorting. The sensitivity can be defined as TP/(TP+FN). True positives (TP) are droplets below the threshold that were correctly selected. False negatives (FN) are droplets with pH values below the threshold, but which were unselected. Likewise, the specificity can be defined as TN/(TN+FP), where true negatives (TN) are droplets above the threshold that were correctly unselected. False positives (FP) are droplets that had pH measurements above the threshold but were selected. These values include only droplets with cells. The large number of empty droplets are correctly sorted to Unselected. For Figure 2C, the sensitivity and specificity were determined to be 94% (66/71) and 91% (48/53), respectively. The values of sensitivity and specificity are comparable to results from previous SIFT devices [12,13] that typically had values of above 90% for both metrics.

It is worth noting that these values of sensitivity and specificity likely overestimate the sorting errors simply due to the variability in the droplet pH values. The false positives and false negatives remain very close to the threshold, within less than 0.05 pH units. This difference is comparable to the variability of the determination of droplet pH, estimated to have a standard deviation of about 0.4 pH units based on the measurement of empty droplets that have an identical pH. Also, as the false positives and false negatives only occur at pH values near the threshold, they do not introduce cells with markedly different glycolytic levels into either the selected or unselected cell populations.

The robust sorting performance is indicative that the travel time required to reach the rail is approximately the same for all droplets. Upon exposure to QX100, droplets gradually acidify, leading to a concurrent increase in interfacial tension. Consequently, a droplet that takes longer to reach the rail may be selected despite having a higher initial pH [20]. As a result, the current device would be expected to exhibit slightly lower specificity compared to earlier systems in which droplets follow identical paths. However, as stated above, a significant decrease in sorting accuracy was not observed. Even if a modest reduction in specificity were present, the several-fold increase in throughput would be an acceptable trade-off for many applications.

Another consequence of the progressive change in interfacial tension as droplets flow through the sorting region is that the threshold depends on the downstream position of the rail [20]. Therefore, for consistency, the rail position was kept in a similar position in the sorting region for the devices in this study. However, in practice, the technique accommodates a range of rail positions, as the entrainment flow can be easily adjusted for a given chip to tune the threshold and obtain the desired fraction of selected droplets.

### 3.2. Increasing Throughput via Parallelization

Parallelization of sorting components has enabled multiplicative gains in throughput for droplet microfluidic applications [33,34]. However, despite its conceptual simplicity, it requires careful design to ensure uniform droplet distribution, consistent operation across sorting elements, and effective collection of common populations. Small changes in dimensions or pressure between sorting elements may result with uneven droplet distribution between sorting elements [30,31,35]. Moreover, with the SIFT technique, different flow rates [12] or relative rail position [20] would also lead to different selection thresholds on individual sorting elements. Overcoming these challenges requires stringent and consistent device microfabrication. The routing of droplets after the sorting elements is also a consideration, as channels are merged to collect common droplet populations [36]. Taken together, the incorporation of parallel sorting components inevitably increases device complexity and the footprint of the device.

SIFT is a passive technique that requires no active sorting components or detectors. As a result, it is particularly well-suited for the parallelization of sorting modules, since it does not increase the overall cost or the number of system components. However, achieving consistent sorting performance across individual sorting elements requires precise design and validation. Previous versions of SIFT devices had a single sorting region; here, we present devices with either two or four sorting regions.

**Figure 3:**
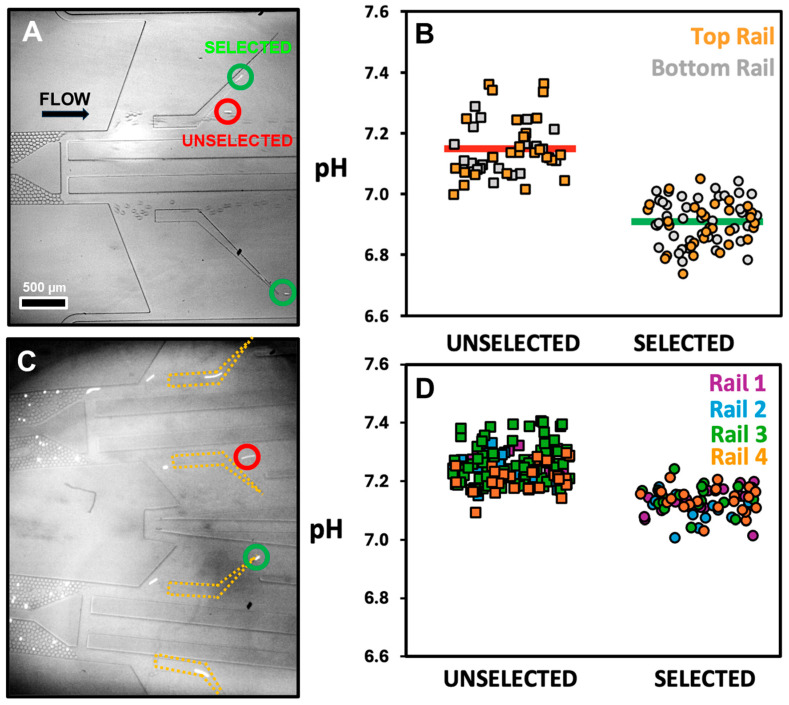
(**A**) Image of sorting region for a device with two sorters. An unselected droplet (circled in red) and selected droplets (circled in green) are highlighted in the image. K562 cells appear as bright spots due to fluorescent labeling. (**B**) pH of droplets containing cells for selected (*N* = 70) and unselected (*N* = 49) populations for the top and bottom rail. Average pH value indicated by horizontal bar. (**C**) Image of sorting region for a device with four sorters. Rails are outlined with dashed yellow lines to enhance visibility in the image. (**D**) pH of droplets containing cells for selected (*N* = 75) and Unselected (*N* = 152) populations for the four rails. Rails are numbered from top to bottom.

Figure 3A and Video S1 demonstrate the geometry of a chip with two sorting regions that are mirror images of each other. The device was designed to permit the observation of both sorting regions within the same imaging field, enabling the simultaneous assessment of sorting metrics for each region. The droplets exiting the incubator are distributed evenly to both sorting regions (see Figure 1B). Immediately prior to entry in the sorting regions, QX100 was introduced in the device via a channel positioned between the two sorting regions (Figure 1B). A QX100 inlet, dubbed the Oil Entrainment Inlet, was positioned in the lateral extremities of the sorting regions. This outlet enabled the control of the oil flow in the sorting region and thus provided a user-defined parameter to adjust the pH threshold. The sorting rail deflects the lateral path of droplets containing cells with high glycolysis. The sorted droplets from the two regions are merged into common selected and unselected outlets, preserving the same number of outlets as in earlier versions of the device.

The throughput of the device was determined to be 100–125 droplets per second for each of the sorting regions or approximately 200–250 droplets per second in total. This represents an increase of about eight-fold from previous chip designs. Figure 3B shows the droplet pH of the two sorting regions. The top and bottom rails exhibit similar thresholds of 7.02 ± 0.01 and 7.04 ± 0.01, respectively (Figure S8). For the bottom rail, the two populations show no overlap, preventing direct determination of the error from the fit. Instead, the uncertainty is reported as the difference between the closest data points of the two populations. This similarity in threshold indicates that the number of sorting regions can be doubled while still selecting the same population of cells. The sensitivity and specificity were high, with values of 99% (68/69) and 96.0% (48/50), respectively. Sensitivity and specificity remain similarly high whether the sorting regions are analyzed together or separately.

Figure 3C and Video S2 show a device with four sorting regions. To avoid expanding the chip width significantly, the sorting region was reduced by half from a lateral width of 1500 µm to 750 µm. Figure 3D shows similar pH thresholds for all four sorting rails, arranged from top to bottom, with pH values of 7.20 ± 0.01, 7.16 ± 0.01, 7.17 ± 0.01, and 7.16 ± 0.01 for rails 1–4, respectively (Figure S9). The sensitivity and specificity were lower than those obtained with the two-sorter device, with sensitivity of 86% (62/72) and specificity of 92% (142/155). The overall sorting throughput of the device with four sorting regions (250 droplets per second) was comparable to that of the device with two sorting regions. The similarity in throughput was likely a result of the reduced channel width that constrained the number of available droplet paths. Additionally, a single-layer device cannot allow the merging of outlets belonging to the same population, whether selected or unselected, into a single outlet. This can only be achieved off-chip or in a two-layer device [36]; however, doing so increases device complexity. Although this design demonstrates the feasibility of further multiplying the sorting regions, the associated complexity and throughput did not justify its use. Consequently, the two-sorting-region device was deemed to be a more practical device; it was thus used for all applications discussed below.

### 3.3. Increasing Cell Collection Through Extended Run Times

The operational stability of microfluidic devices over extended run times, though often unreported, is a critical performance metric for practical applications. As an inline device, the number of sorted cells is proportional to the overall run time. However, this requires a device that is stable over time, with a steady input of cells. A particular challenge that is associated with long run times is the tendency of cells to sediment in the syringe and tubing before reaching the microfluidic device. This sedimentation is exacerbated by the low flow rates of the cell suspension into our device (typically 0.3 μL/min, Table S1). A density modulator (OptiPrep) is used to match cell and solution densities. While this reduces cell sedimentation, the number of cells entering the device decreases substantially after approximately 20 min. Optimizing the concentration of Optiprep can help alleviate this problem but does not eliminate it.

To ensure a constant flow of cells over the entire device run time, a small stir bar was placed inside the syringe containing the cell solution. About every ten minutes, the magnetic stir bar inside the syringe was moved back and forth using a larger external magnet to resuspend the cells. Furthermore, PEEK tubing with a small inner diameter (0.18 mm) was used to connect the syringe to the device. The small inner diameter limited cell sedimentation during the residence time in the tubing (approximately 25 min). Together, these modifications maintained a consistent influx of cells into the device, allowing for experiments of extended duration.

**Figure 4:**
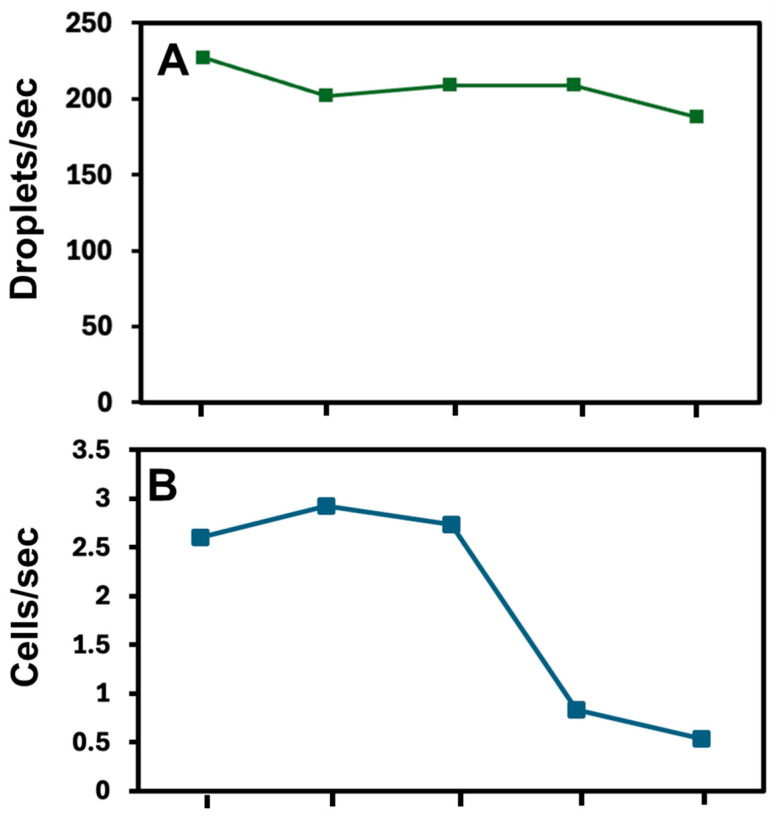

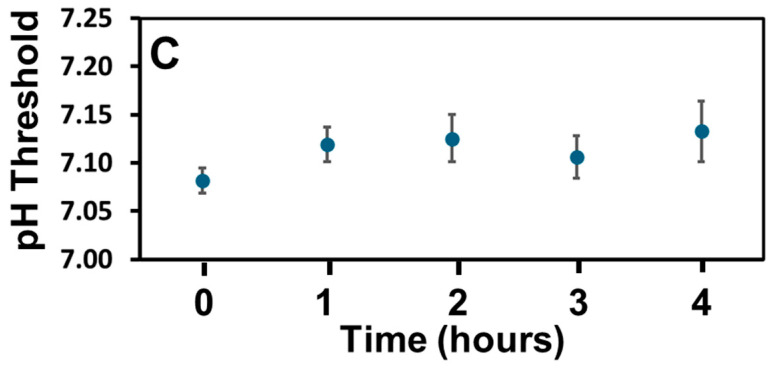
Sorting rate and threshold for a continuous run on a two-sorter device. To obtain sufficient measurements, each data point represents the analysis of two 2 min videos acquired 10 min apart. (**A**) Rate of droplet sorting at different time points for the SIFT device. (**B**) Rate of cell sorting. (**C**) pH threshold of sorting. Error bars represent standard error.

Figure 4 summarizes the temporal stability of the device, based on measurements obtained at discrete time points over several hours. In this experiment, the entrainment and oil outlet flow rates were adjusted three times in the first hour to maintain a consistent fraction of selected droplets (Table S2). As shown in Figure 4A, the droplet-sorting rate remains stable at approximately 200–225 droplets per second over the entire 4 h run time. Figure 4B shows the number of cells sorted per second at different time points, revealing a decrease in cell entry after approximately 2 h of operation. The decrease in cell entry after two hours may result from gradual cell sedimentation, aggregation, or subtle changes in viability during extended run times. Despite this reduction, the pH sorting threshold remains consistent over the 4 h (Figure 4C and Figure S10). The sensitivity and specificity for the run were 92% (110/120) and 96% (358/372), respectively. In summary, although the threshold and droplet-sorting rate are consistent over the 4 h, the practical run time is ultimately constrained by the rate of cell entry into the device. These results indicate an operational run time of approximately 2 h for the SIFT device. For long runs, the device still requires intermittent user intervention to remove oil in the collection pipettes and to resuspend the cells in the syringe. Occasional adjustments to the flow rates helped maintain a consistent fraction of sorted cells. This adjustment is likely needed to correct for subtle pressure drifts in the device or a gradual decrease in the bulk pH of the cellular solution. The long run time shown here is critical to collect a large number of sorted cells for many biological applications.

### 3.4. Application to Cellular Iron Uptake

As a demonstration of the sorting capacity of the device and its integration into a biological assay workflow, the relationship between glycolysis and iron uptake was explored in activated Jurkat T cells. Iron plays an important role in T cell proliferation and differentiation, in part through its involvement in mitochondrial metabolism [37]. Iron supports T cell activation by enabling mitochondrial respiration, anabolic metabolism, and redox-dependent signaling [37,38]. Activated T cells upregulate iron uptake to meet the increased metabolic demands associated with proliferation and effector differentiation [23]. As activated T cells differentiate to perform specific cellular functions, iron availability and regulation influence functional outcomes. Previous work has shown that iron uptake drives T cells into a proinflammatory phenotype via sped-up metabolic rates [23]. In contrast, hypoferremia, or low iron, counteracts the development of adaptive immunity [39]. However, the underlying mechanisms by which iron regulation shapes metabolic heterogeneity and functional diversity at the single-cell level remain poorly understood. This motivates approaches that can directly link iron uptake to metabolic state in individual cells.

**Figure 5:**
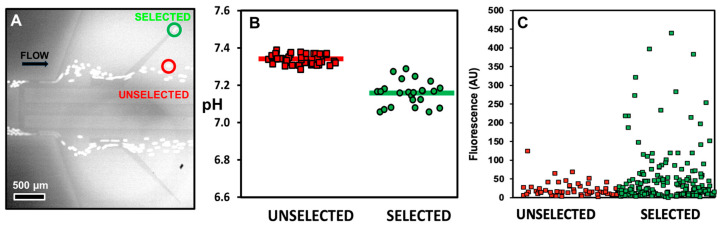
(**A**) Image of sorting region showing an unselected droplet (circled in red) and a selected droplet (circled in green). (**B**) pH of selected (*N* = 24) and unselected (*N* = 50) cell populations. Average pH value indicated by horizontal bar. (**C**) Maximum cellular fluorescence intensity of BioTracker Far-red Labile Fe^2+^ Live Cell Dye for the unselected and selected cell populations. The selected (*N* = 192) and unselected populations (*N* = 61) were statistically different (Welch’s *t*-test, (t(148.1) = −3.13, *p* = 0.002).

Prior to sorting, activated Jurkat T cells were incubated with a fluorescent iron sensor (BioTracker Far-red Labile Fe^2+^ Live Cell Dye). Figure 5A shows the sorting of droplets containing activated T cells using the device presented earlier containing two sorting regions. Selected and unselected droplets are indicated in green and red, respectively. The overall throughput was about 240 droplets per second, and the sorted droplets were collected for 120 min. Figure 5B shows the pH of individual droplets of the selected and unselected droplets. The pH threshold was determined to be 7.29 (Figure S11). No error is reported because the two populations do not overlap; they only share a common boundary value, preventing us from making a well-defined estimate of uncertainty. Based on the pH measurements in Figure 5B, both the sensitivity and specificity were 100%. These experiments were performed on cells without a viability marker to avoid spectral overlap with the fluorescent iron sensor. It is thus difficult to confirm the presence of a cell in a droplet in videos. Hence, the high values of sensitivity and specificity may be partly attributed to the inclusion of empty droplets within the measurements of the unselected population. Following sorting, cells were recovered by droplet coalescence using a protocol (Figure S5) described in Shulman et al. [20] and imaged on a fluorescent microscope. Max cell intensity rather than the cellular average was measured as the labile iron dye is expected to be localized in the endoplasmic reticulum. Figure 5C shows the BioTracker single-cell fluorescent intensity of the selected and unselected populations. While some selected highly glycolytic cells share similar iron uptake to low glycolytic cells, a significant number of highly glycolytic cells show increased iron uptake. Selected cells exhibited a 2.27-fold higher maximum cell intensity compared to the unselected population. The Welch’s two-sample *t*-test confirmed a significant difference between the two populations. The same conclusion was derived from a replicate dataset (Figure S12). Welch’s test was used because the populations exhibited unequal variances. As single-cell measurements often produce skewed distributions due to rare high-intensity events, the data was log-transformed prior to statistical analysis to better approximate normality. Overall, Figure 5 reveals that glycolysis and iron homeostasis are correlated for a subset of cells. This experiment brings a new approach of examining this relationship for individual cells. It enables the exploration of cells that are far from the average. This association can be further explored at the single-cell level to elucidate potential mechanisms underlying the correlation between glycolysis and iron uptake.

## 4. Conclusions

A systematic improvement of the throughput and run time of the SIFT device is presented. The use of smaller droplets that take a breadth of sorting paths enabled a substantial increase in the number of droplets treated by a single sorting element. This change allowed an increase in throughput of approximately a factor of four from 30 droplets per second to 125 droplets per second. Sorting throughput is further increased by parallelization, and the use of two and four independent sorting regions were demonstrated. These devices distribute the droplets within the sorting elements. The elements were shown to display similar pH sorting thresholds. As drag force is a determinant component in sorting, this indicates similar flow conditions are achieved in the different sorting regions. The device with two sorting elements was found to offer best results with respect to throughput, robustness and simplicity. The sorted populations from the two sorting elements can be easily combined at the outlet. This device had a maximum throughput of about 250 droplets per second. In many biological applications, only one in 20–30 droplets are occupied to minimize multiple-cell occupancy, resulting in an effective sorting rate of approximately 8 cells per second. By minimizing cell sedimentation in the syringe and tubing this device was shown to be stable for 2 h of run time. The two-sorter device exhibited reproducibility with similar sorting metrics (throughput, sensitivity, specificity) over multiple runs on different devices with the same geometry (Table S3). Notably, because the system relies on a single device, preparation requires only about 30 min, after which it can be operated for extended periods to sort the number of cells needed for the desired application. A comparison of the sorting metrics of the two and four sorting region devices as compared to the previous devices is presented in Table S4. The two-sorting region device performs better than, or comparable to, earlier devices across all studied metrics.

Further improvements in throughput are likely possible. Higher throughputs may be possible by using even smaller droplets; however, optimal flow and rail position would be needed to maintain reliable sorting. Droplet interfacial tension increases as they flow downstream from exposure to QX100 solution. Therefore, to achieve faster throughputs, the rail could be positioned further downstream than presented here. A massively parallel architecture is also conceivable. However, implementing such a design is not trivial due to constraints inherent to the SIFT device. In particular, the QX100 solution can only be introduced immediately prior to sorting, which complicates the device layout and limits how parallelization can be implemented.

The cell sorting rate is also limited by the fact that most sorted droplets are empty to avoid multiple occupancy. If the biological application can tolerate multiple cell occupancy the effective cell sorting rate can be increased by utilizing higher cell densities. The literature includes both active and passive methods for deterministic single-cell encapsulation, ensuring that each droplet contains a single cell [40,41]. In particular, the use of inertial ordering is an effective passive method to populate droplets with a single cell. However, the very high flow rates required for this technique render it incompatible with the incubation step of SIFT within a single device. It could be used if the encapsulation is performed on a separate device. Droplets would then be re-injected after incubation into a sorting device. That particular workflow would not preclude the advances in throughput presented here; the improvements could also be adapted to a dedicated sorting device.

The improved device was integrated into a biological workflow. Cells were sorted, recovered, and subsequently analyzed to study the relationship between cell glycolysis and iron homeostasis at the single-cell level. The results show that there is a subset of cells that display significantly higher iron uptake within the highly glycolytic cells. These findings support previous research of the interplay between iron homeostasis and T cell metabolism [38]. This work brings a novel approach to examining iron homeostasis through the lens of single-cell metabolism. The performance improvements significantly expand the investigative capacity of the SIFT technique for biological studies requiring greater scale or the capture of rare metabolically distinct cells.

## Data Availability

The original contributions presented in this study are included in the article. Further inquiries can be directed to the corresponding author.

## Supplementary Materials

The following supporting information can be downloaded at: https://www.mdpi.com/article/10.3390/mi17060691/s1, Figure S1: SIFT device with two sorting regions channel geometry; Figure S2: Sorting area dimensions for the device with two sorting regions; Figure S3: SIFT device with four sorting regions channel geometry; Figure S4: Sorting area dimensions for the device with four sorting regions; Figure S5: Workflow for cell collection; Figure S6: Logistic regression fit for all lateral positions; Figure S7: Logistic regression fit for different lateral positions; Figure S8: Logistic regression fit for top and bottom Rail; Figure S9: Logistic regression fit for rails 1-4; Figure S10: Logistic regression fit at discrete time points; Figure S11: Logistic regression fit of activated Jurkat T cells; Figure S12: Fluorescence intensity of BioTracker for Unselected and Selected cell populations; Table S1: Typical flow parameters for a two-sorting region device; Table S2: Flow parameters for each figure; Table S3: Two Sorting Region Reproducibility; Table S4: Comparison of SIFT Sorting Device Metrics; Video S1: SIFT device with two sorting regions; Video S2: SIFT device with four sorting regions.
