# Supplementary material for: Optimization and Parallelization of Sorting by Interfacial Tension (SIFT) for High-Throughput Metabolic Cell Sorting
Source: Micromachines (Basel). 2026 Jun 3;17(6):691. doi: 10.3390/mi17060691 (PMC13303160; doi:10.3390/mi17060691)
Supplement: Supplementary file 1 [file micromachines-17-00691-s001.zip › Optimization and Parallelization of Sorting SI.pdf]

Optimization and Parallelization of Sorting by Interfacial Tension (SIFT) for High-Throughput Metabolic Cell Sorting

Aria Trivedi[1], Thomas Mathew[1], Matthew Shulman[1], Lakshmi Thangam[1], Pooja Dubey[1], Charlotte V. Cohen[1], Kelsey Voss[2], Paul Abbyad[1*]

1 Department of Chemistry and Biochemistry, Santa Clara University, Santa Clara, CA, 95053, USA
2 Department of Pharmacology, University of Virginia, Charlottesville, VA, 22903, USA * corresponding author: pabbyad@scu.edu

Supplemental Information

Table of Contents:

Video Caption……………………………………………………………..…………..…..Page S-2

Figure S1…...…………………………………………………………………….…..…..Page S-3

Figure S2…...…………………………………………………………………….…..…..Page S-4

Figure S3…...…………………………………………………………………….…..…..Page S-5

Figure S4…...…………………………………………………………………….…..…..Page S-6

Figure S5…...…………………………………………………………………….…..…..Page S-7

Figure S6…...…………………………………………………………………….…..…..Page S-8

Figure S7…...…………………………………………………………………….…..…..Page S-9

Figure S8…...…………………………………………………………………….…..…..Page S-10

Figure S9…...…………………………………………………………………….…..…..Page S-11

Figure S10...…...…………………………………………………………….…...…..Page S-12

Figure S11…...………………………………………………………………...…..…..Page S-13

Figure S12…...………………………………………………………………...…..…..Page S-14

Table S1…......…………………………………………………………………………..Page S-15

Table S2…......…………………………………………………………………………..Page S-16

Table S3…......…………………………………………………………………………..Page S-17

Table S4…......…………………………………………………………………………..Page S-18

Video Caption:

Video S1. SIFT device with two sorting regions:
In this video, two sorting regions are used to increase droplet sorting throughput. K562 cells appear as bright spots due to fluorescent labeling. Droplets with cells with high glycolysis are deflected laterally to the Selected outlet. Empty droplets or those containing cells with low glycolysis are only slightly deflected by the rail and are directed to the Unselected outlet. Video slowed by 3X.

Video S2. SIFT device with four sorting regions:
In this video, four sorting regions are used to increase droplet sorting throughput. K562 cells appear as bright spots due to fluorescent labeling. Video slowed by 3X

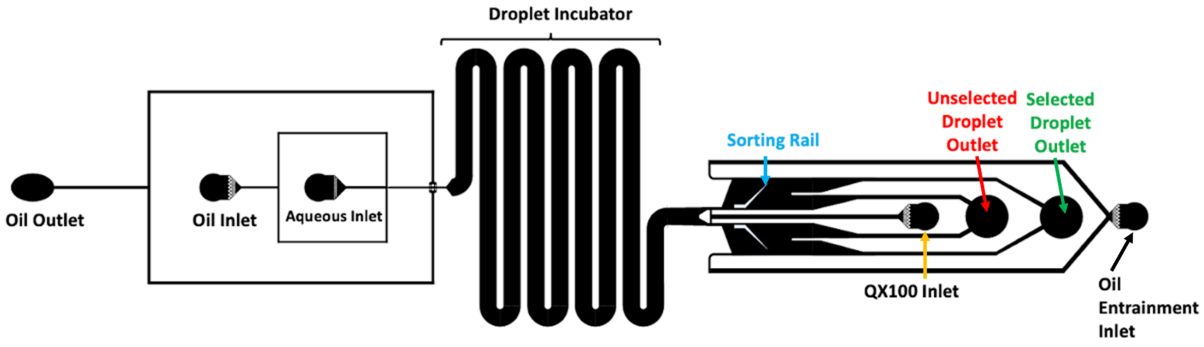

Supplemental Figure S1. SIFT device with two sorting regions channel geometry.

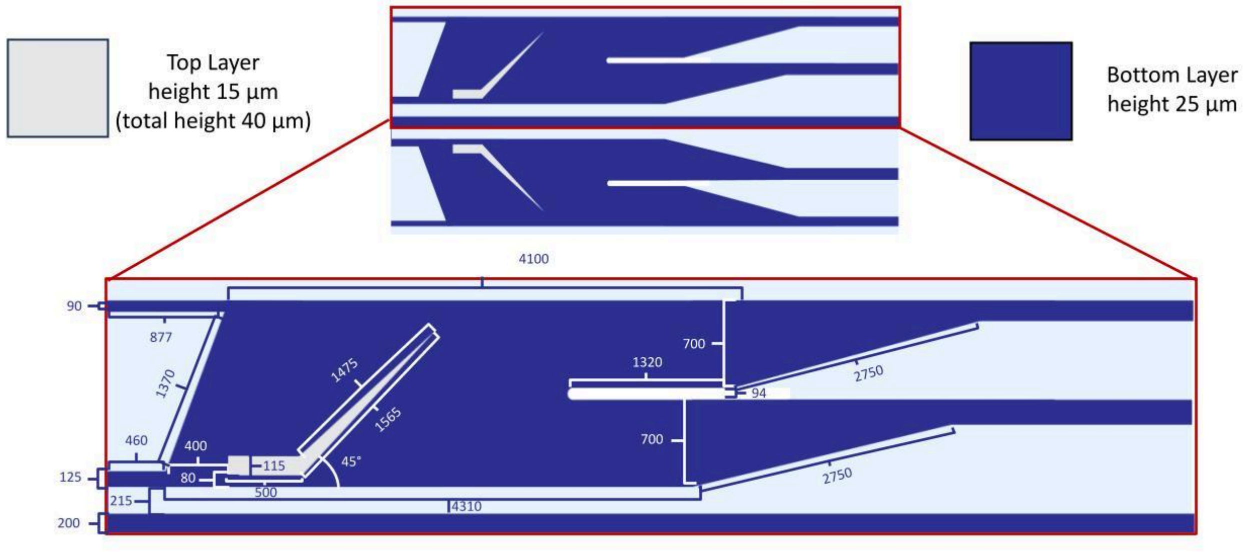

Supplemental Figure S2. Sorting area dimensions for the device with two sorting regions. One set of sorting area measurements are shown as both sorting areas have the same dimensions. The rail was positioned under a microscope during the microfabrication process using alignment markers. The relative position of the rail in the channel was about ±70 microns in both the vertical and horizontal direction.

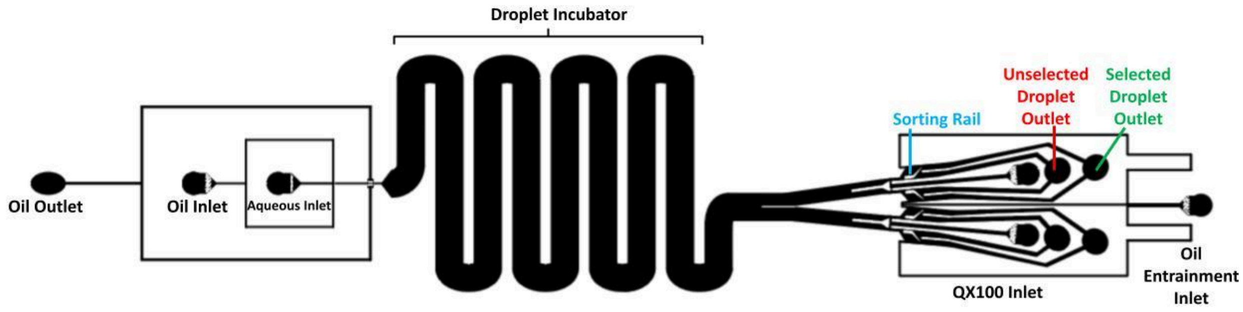

Supplemental Figure S3. SIFT device with four sorting regions channel geometry.

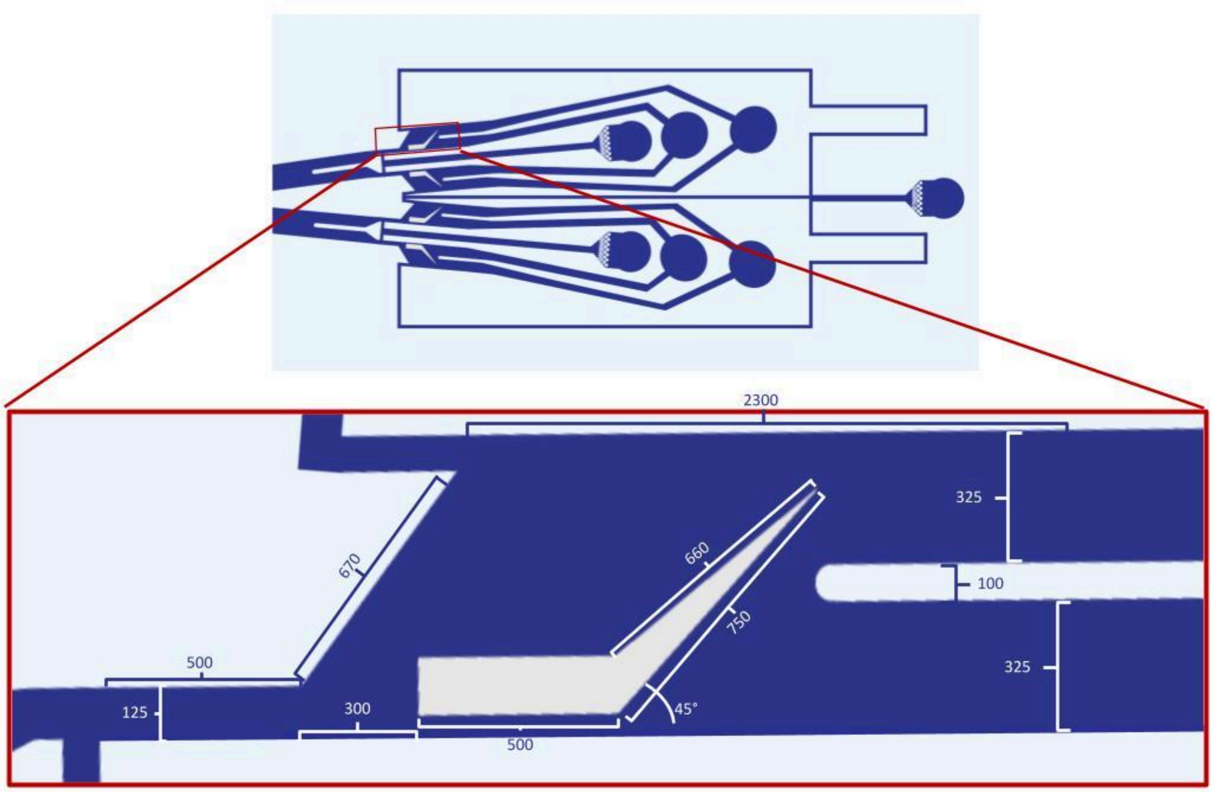

Supplemental Figure S4. Sorting area dimensions for the device with four sorting regions. One set of sorting area measurements are shown as all sorting areas have the same dimensions. The rail was positioned under a microscope during the microfabrication process using alignment markers.

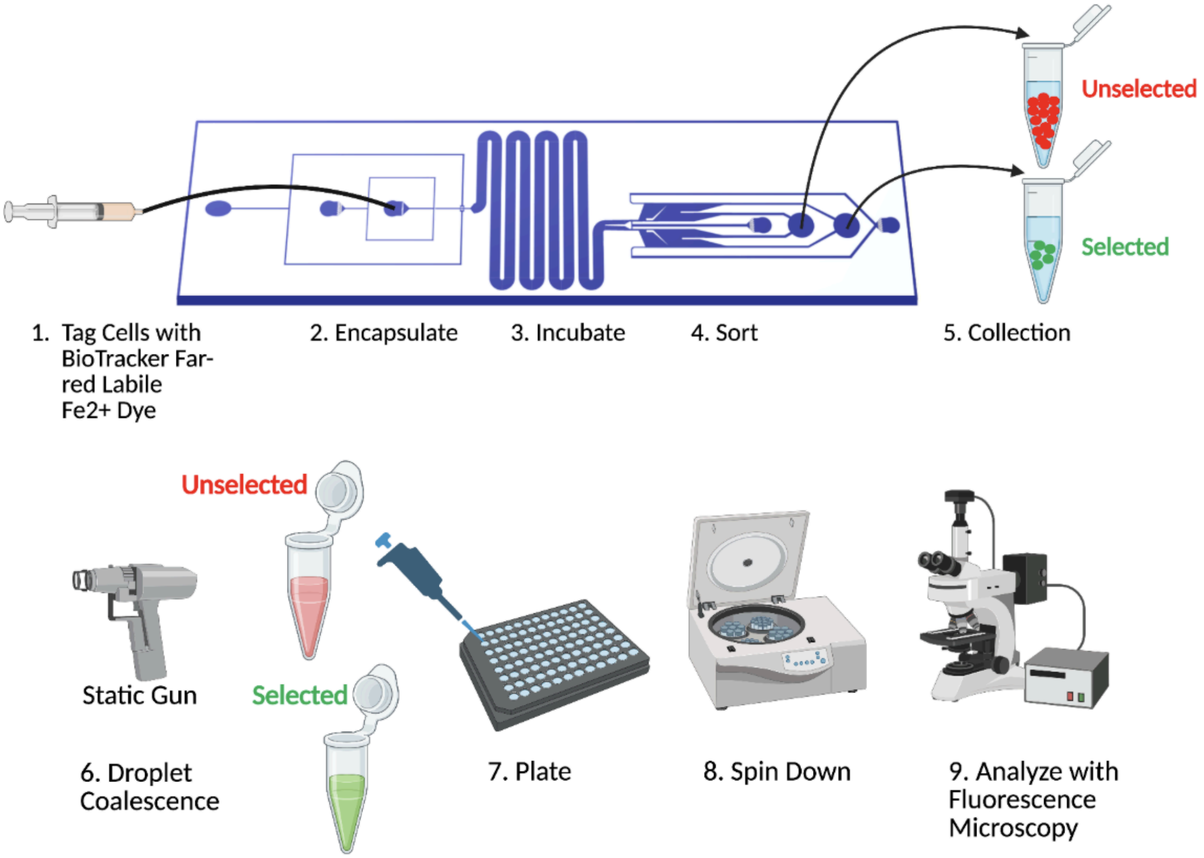

Supplemental Figure S5. Workflow for cell collection. Created in BioRender.com.

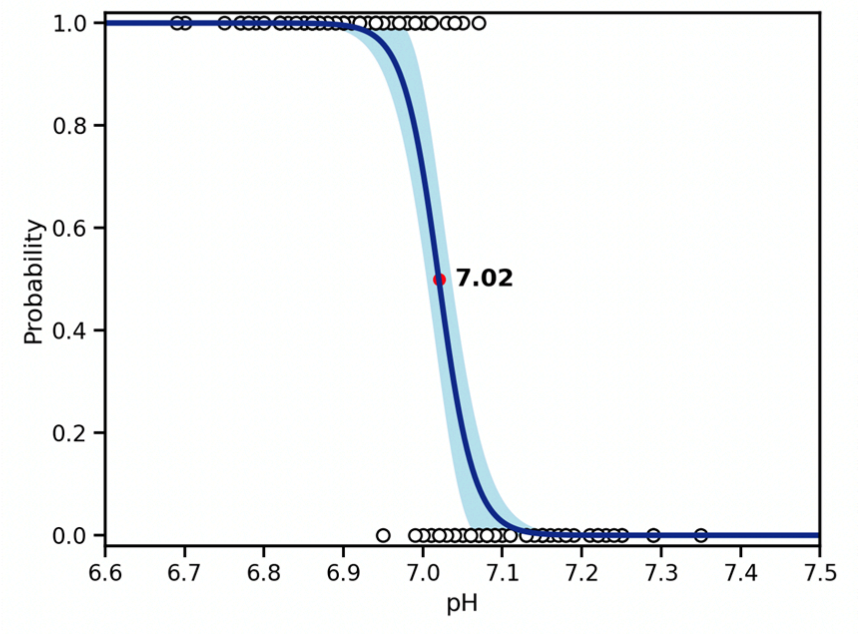

Supplemental Figure S6. Logistic regression fit of binary Selected/Unselected data (*N*=124) with pH. pH thresholds are indicated on the graph and represent where there is equal probability that droplets are Selected or Unselected. The 95% confidence limit is indicated in light blue.

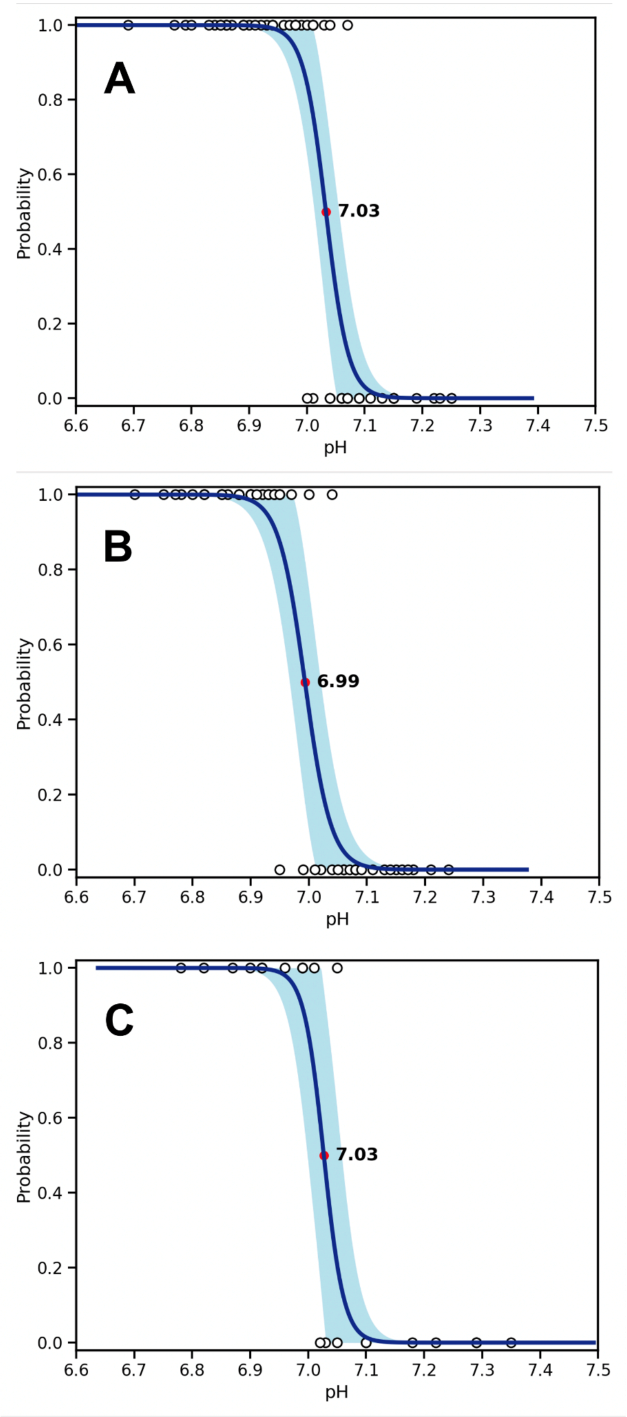

Supplemental Figure S7. Logistic regression fit of binary Selected/Unselected data with pH for a (**A**) maximum lateral position 0–50 µm (*N*=54) (**B**) 50–100 µm (*N*=49) (**C**) >100 µm (*N*=21).

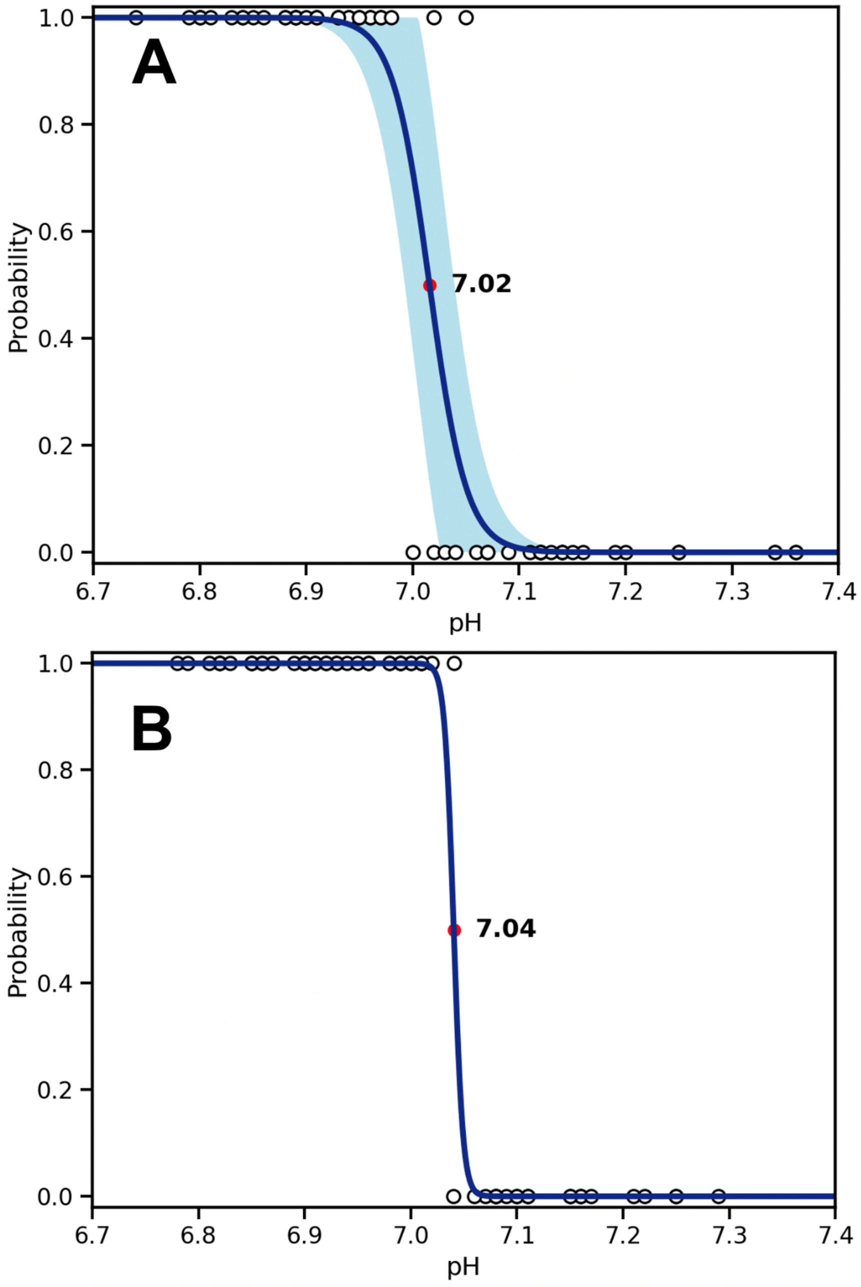

Supplemental Figure S8. Logistic regression fit of binary Selected/Unselected data with pH for a device with two sorting regions. (**A**) Top Rail (*N*=59) (**B**) Bottom Rail (*N*=60). No error in threshold is obtained from the fit as the two populations show no overlap.

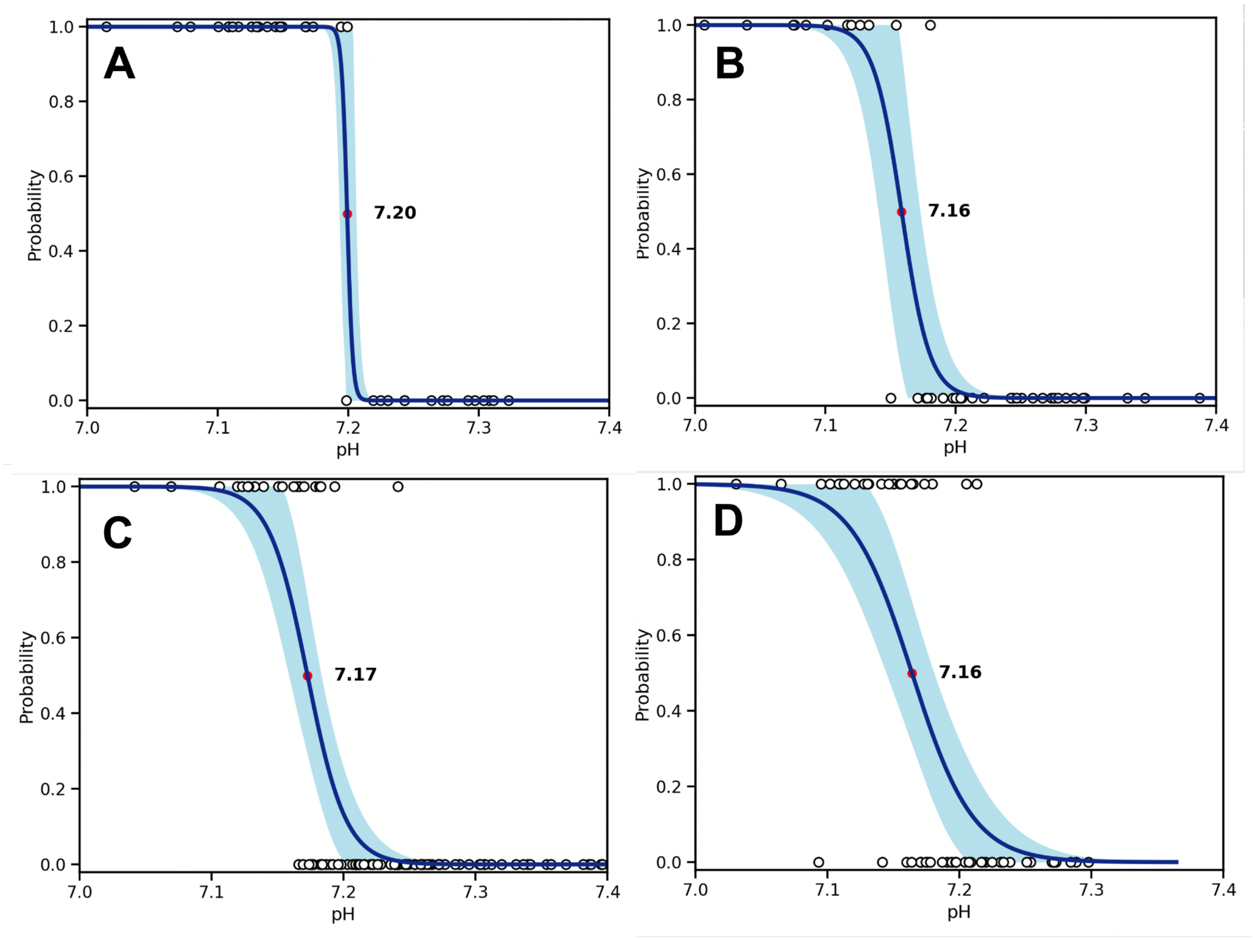

Supplemental Figure S9. Logistic regression fit of binary Selected/Unselected data with pH for a device with four sorting regions. (**A**) Rail 1 (*N*=37) (**B**) Rail 2 (*N*=41) (**C**) Rail 3 (*N*=94) (**D**) Rail 4 (*N*=55).

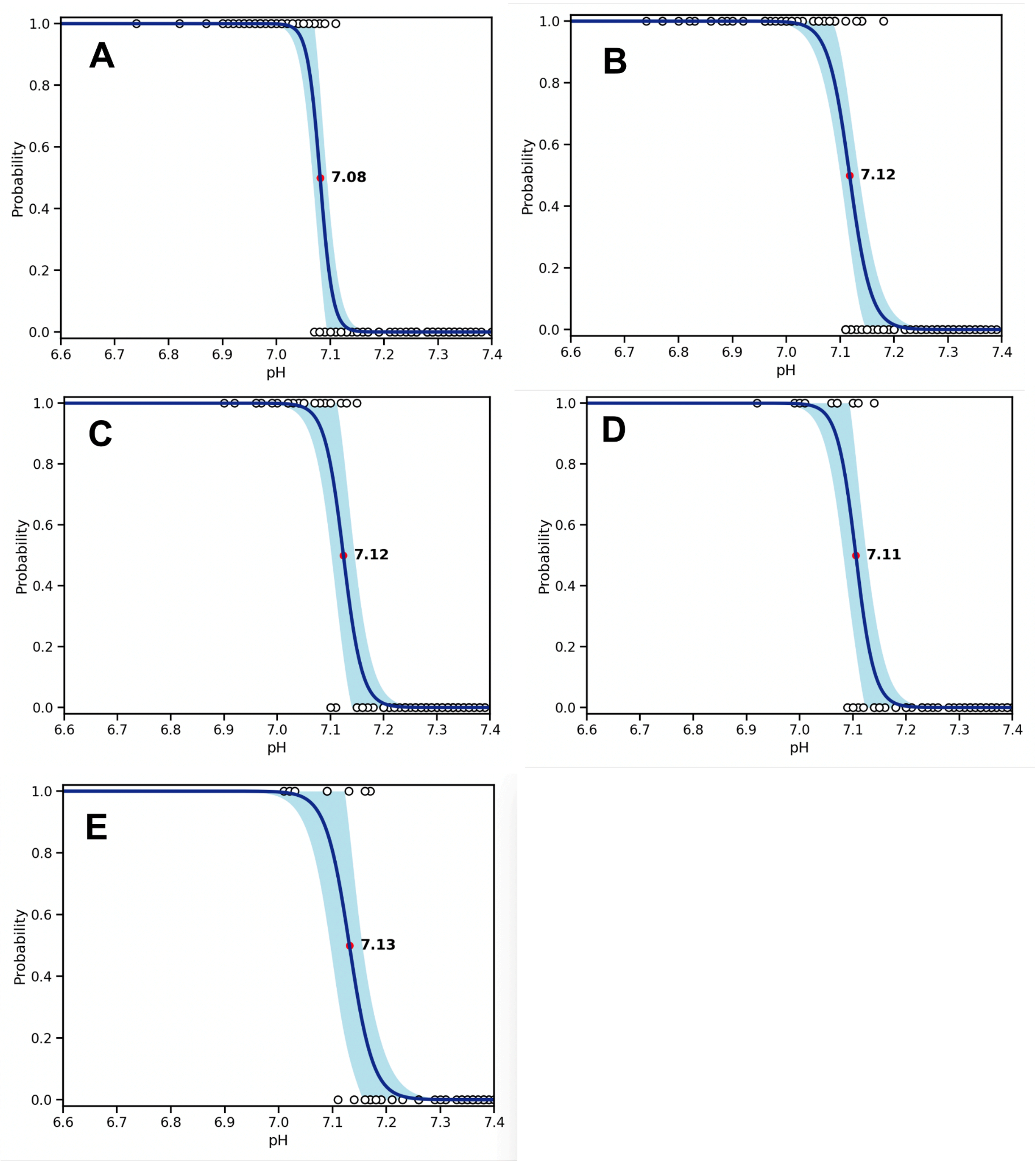

Supplemental Figure S10. Logistic regression fit of binary Selected/Unselected data with pH at discrete time points. (**A**) 0 hour (*N*=136) (**B**) 1 hour (*N*=130) (**C**) 2 hours (*N*=103) (**D**) 3 hours (*N*=66) (**E**) 4 hours (*N*=57).

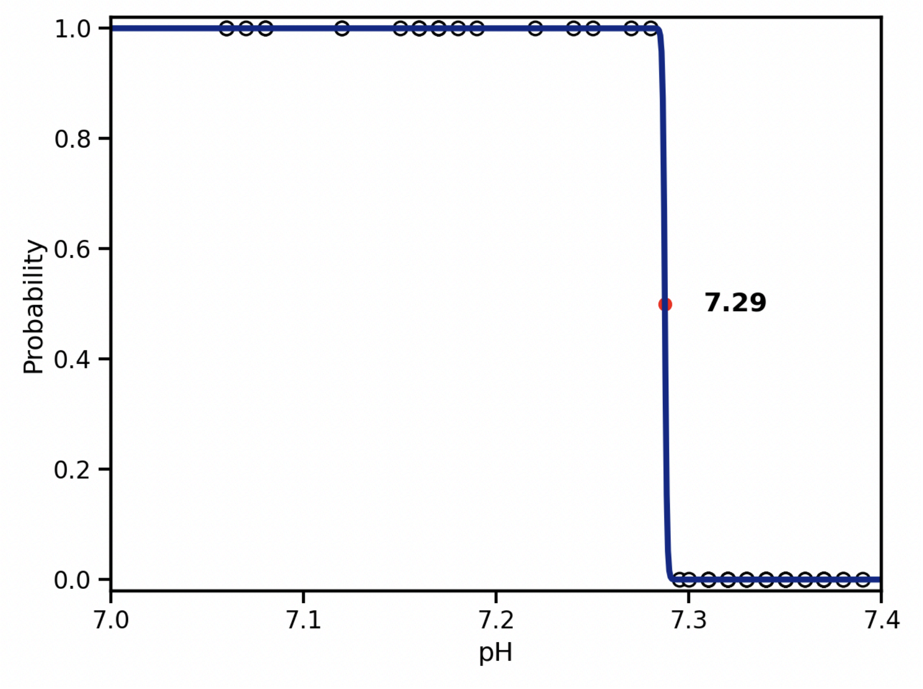

Supplemental Figure S11. Logistic regression fit of binary Selected/Unselected data (*N*=74) with pH for activated Jurkat T cells. No error in threshold is obtained from the fit as the two populations do not overlap and only share a common boundary value, preventing a well-defined estimate of threshold uncertainty.

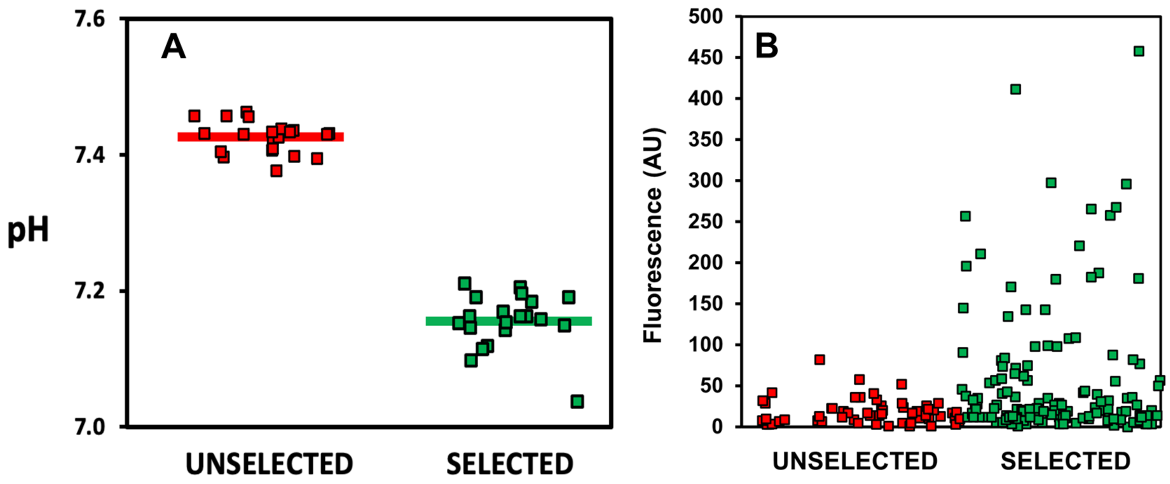

Supplemental Figure S12. a) pH of Selected (*N* = 20) and Unselected (*N* = 21) cell populations. Average pH value indicated by horizontal bar. b) Maximum cellular fluorescence intensity of BioTracker Far-red Labile Fe^{2+} Live Cell Dye for the Unselected and Selected cell populations. The Selected (*N* = 143) and Unselected populations (*N* = 58) were statistically different (Welch's t-test, (t(5.11) = −151.9, p = 9.5e-07).

Supplemental Table S1. Typical flow parameters for a two-sorting region device. Channel geometry is provided below for reference. Negative flows below are opposite in direction to the main flow in the channel.

Inlets and Outlets	Flow Rates (μL/min)
Aqueous Inlet	0.3
Oil Inlet	3
QX100 Inlet	10
Oil Entrainment Inlet	35 – 49
Oil Outlet	- 2 to -2.8

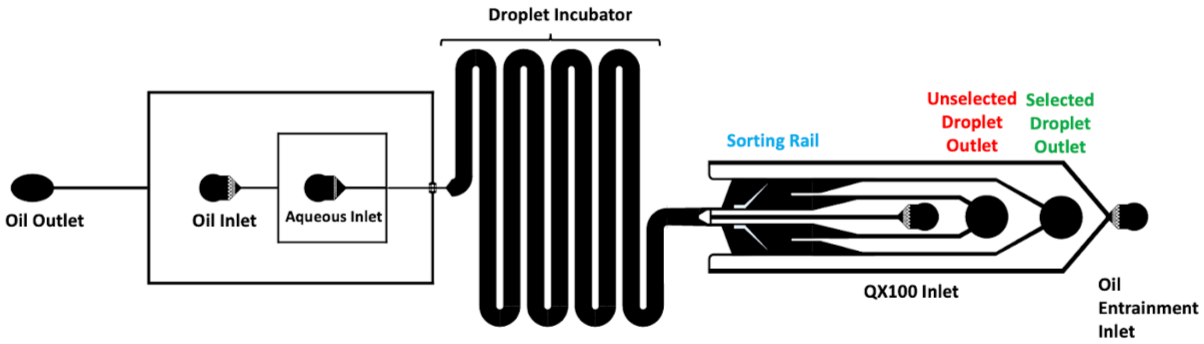

Supplemental Table S2. Flow parameters for each figure. Negative flows below are opposite in direction to the main flow in the channel.

Figure #	Chip Design	Flow Rates (µL/min)				
		Aqueous Inlet	Oil Inlet	QX100 Inlet	Entrainment Inlet	Oil Outlet
2	Two-Sorting Regions	0.3	3	10	48	-3
3A/B	Two-Sorting Regions	0.3	3	10	48	-3
3C/3D	Four-Sorting Regions	0.3	3	10	45	-2.5
4 *	Two-Sorting Regions	0.3	3	10	40 - 50	-2 to -2.7
5 **	Two-Sorting Regions	0.3	3	10	35 - 45	-2

* The flow rates were adjusted three times during the first hour to maintain consistent fractional selection of droplets.

** The flow rates were adjusted intermittently (approximately every 20–25 minutes) to maintain a consistent fraction of selected droplets.

Supplemental Table S3. Two Sorting Region Reproducibility. Runs performed on different devices with the same channel geometry.

Run	Cell Type	Throughput (droplets/ sec)	Threshold	Threshold Top Sorter	Threshold Bottom Sorter	Difference in Threshold	Sensitivity	Specificity
1	K562	250	7.03	7.02	7.04	0.02	99% (68/69)	96% (48/50)
2	K562	200	7.11	7.12	7.10	0.02	92% (110/120)	96% (358/372)
3	K562	270	6.88	6.90	6.87	0.03	82% (27/33)	92% (70/76)
4	Jurkat *	265	7.29	7.30	7.22	0.08	100% (24/24)	100% (50/50)
5	Jurkat *	200	7.26	7.30	7.26	0.04	100% (20/20)	100% (37/37)
6	Jurkat *	225	7.23	7.26	7.24	0.02	100% (23/23)	100% (52/52)

* High values of sensitivity and specificity may be partly attributed to the inclusion of empty droplets within the measurements of the Unselected population

Supplemental Table S4. Comparison of SIFT Sorting Device Metrics.

	Previous SIFT devices (Zielke, 2020; Zielke, 2022)	Device with two Sorting Regions	Device with four Sorting Regions
Chip Preparation Time (min)	30	30	30
Droplet Size (µm)	70 - 90	40	40
Incubation Time (min)	6-8	3 - 6	3 - 6
Number of Sorting Regions	1	2	4
Max Droplet Sorting Throughput (droplets/sec)	30	250	250
Max Cell Sorting Throughput (cells/sec) *	1	8	8
Sensitivity (%)	>90	>90	>85
Specificity (%)	>90	>90	>90
Max Operational Runtime (min)	20	120	Not determined
Viability (%) **	50	50	50

* Assume cell occupancy of one cell per 30 droplets.

** Viability was not directly measured here but was determined in a previous publication using a similar device and workflow (Shulman, *Lab Chip*, 2026). A control population of cells not sorted on the device exhibited a viability of 70%.
